# A holder-type plasma cleaner for in-situ removal of hydrocarbon contamination in the transmission electron microscope chamber

**DOI:** 10.1186/s42649-026-00138-6

**Published:** 2026-05-20

**Authors:** Hang Sik Kim, Hyun-Woong Park, Pan Kyu Kim, Myung Jae Lee, Hong Kim, Yong Kim, Young-Min Kim

**Affiliations:** 1 Department of Energy Science, Sungkyunkwan University (SKKU), Suwon, 16419 Republic of Korea; 2 INTEC Corporation Co, Ltd, Suwon, 16544 Republic of Korea; 3 Center for 2D Quantum Heterostructures, Institute for Basic Science (IBS), Suwon, 16419 Republic of Korea

**Keywords:** Scanning transmission electron microscopy (STEM), Hydrocarbon contamination, Plasma cleaning, Holder-type plasma cleaner, In-situ chamber cleaning

## Abstract

Hydrocarbon contamination is a common problem in transmission electron microscopy (TEM), affecting image contrast and the accuracy of quantitative spectroscopic analysis. Although plasma cleaning of specimen holders and samples is widely used, fully removing hydrocarbon contaminants from inside the microscope chamber has mostly involved slow processes like column baking, which can take several days of instrument downtime. In this study, we introduce a holder-type plasma cleaner that can be inserted directly into the TEM chamber through the specimen port. The device produces oxygen-radical plasma powered by radio frequency (RF) at 13.56 MHz, allowing in-situ cleaning of internal chamber surfaces in around three hours. We evaluated the cleaning performance quantitatively using scanning TEM (STEM)-energy-dispersive X-ray spectroscopy (EDX) and electron energy-loss spectroscopy (EELS). After one cleaning cycle, the carbon-K signal increase rate dropped from 2.81 to 0.30%/frame, and the carbon deposition rate decreased from 0.54 to 0.02 nm/frame, roughly a tenfold decrease. Repeated cleaning after three months of routine use further lowered contamination rates. These test results show that holder-type plasma cleaning is an effective, time-saving alternative for regular maintenance of TEM chamber cleanliness.

## Introduction

Hydrocarbon contamination remains one of the most persistent obstacles in transmission electron microscopy (TEM). Under electron-beam irradiation, residual hydrocarbon molecules adsorbed on specimen surfaces or present within the microscope chamber undergo radiolysis and polymerization, accumulating as amorphous carbonaceous deposits (Egerton et al. 2004). These deposits degrade image contrast, obscure fine structural details, and compromise the reliability of quantitative spectroscopic analysis (Kim et al. 2021a, b; Yang et al. 2021). The problem is particularly acute in modern aberration-corrected scanning transmission electron microscopy (STEM), where highly focused sub-angstrom probes concentrate the electron flux into extremely small areas, accelerating the rate of local carbon buildup by orders of magnitude compared to conventional parallel-beam illumination (Hugenschmidt et al. 2023).

Contamination originates from multiple sources, including residual hydrocarbons introduced during specimen preparation, outgassing from holder components and O-ring seals, vacuum grease residues, and hydrocarbon species adsorbed on internal microscope surfaces. Even commercial carbon support films have been shown to carry substantial mobile hydrocarbon species (McGilvery et al. 2012; Hettler et al. 2017). A variety of strategies have been developed to mitigate these effects, including ultrasonic cleaning of components, plasma cleaning of holders and specimens, cold traps using liquid nitrogen or Peltier cooling, column baking, beam showering, and ultraviolet-ozone treatment (Mitchell 2015; Isabell et al. 1999; He 2023). Among these, plasma cleaning is the most widely adopted because it effectively removes hydrocarbon species through oxidation by reactive radicals without requiring mechanical disassembly (Isabell et al. 1999; Li et al. 2021).

Current commercial plasma cleaning systems for electron microscopy fall into two main categories. External benchtop systems, such as the Fischione Model 1020/1070 and Gatan Solarus II, clean specimens and holders in a dedicated vacuum chamber before insertion into the microscope. While effective for removing surface contamination from holders, these systems cannot access hydrocarbon reservoirs that reside on internal chamber surfaces (Goh et al. 2020). Remote plasma sources, exemplified by the Evactron series, mount externally on microscope ports and deliver reactive oxygen radicals into the chamber through downstream diffusion (Levesque 2011). Although these devices can reduce contamination levels during routine operation, they are typically installed on external ports, and the plasma source is positioned at some distance from the specimen chamber interior. The holder-type plasma cleaner described in this work provides a complementary approach by positioning the plasma source directly inside the chamber, offering a practical alternative for TEM systems without a built-in or externally mounted chamber-cleaning system. Tang et al. (2023) recently demonstrated an integrated apparatus combining plasma cleaning with vacuum storage for holders and specimens; however, this system also operates externally rather than inside the TEM column. Despite these advances, contamination originating from within the TEM chamber itself remains difficult to address effectively. Internal components, including the chamber walls, condenser and objective apertures, and the pole-piece region, accumulate hydrocarbon species over time and act as persistent reservoirs that continuously replenish surface contamination (Tang et al. 2023; Mitchell 2015). Conventional maintenance procedures such as cold-trap reconditioning and column baking can reduce these internal contaminants without disassembly, but they are time-consuming and operationally disruptive, often requiring one to several days of microscope downtime. As a result, there is a practical need for a cleaning method that can directly access and decontaminate the internal chamber environment with substantially shorter turnaround time.

In this report, we demonstrate a holder-type plasma cleaner designed to be inserted directly into the TEM chamber through the specimen holder port. The device generates oxygen-radical plasma under radio frequency (RF) excitation at 13.56 MHz, enabling in-situ cleaning of contamination sources within the microscope column in approximately three hours. Unlike external plasma cleaners that treat only the holder and specimen, the present approach positions the plasma source inside the chamber, allowing reactive species to reach internal surfaces that are otherwise inaccessible without full disassembly. The cleaning effectiveness was quantitatively evaluated using STEM-energy-dispersive X-ray spectroscopy (STEM-EDX) and electron energy-loss spectroscopy (EELS). Two complementary contamination metrics were employed: the C-K signal increase rate and the carbon deposition rate measured before cleaning, after cleaning, and over several months of routine microscope operation to assess both the immediate effect and the long-term persistence of the treatment.

## Materials and methods

### Holder-type plasma cleaning procedure

**Fig. 1:**
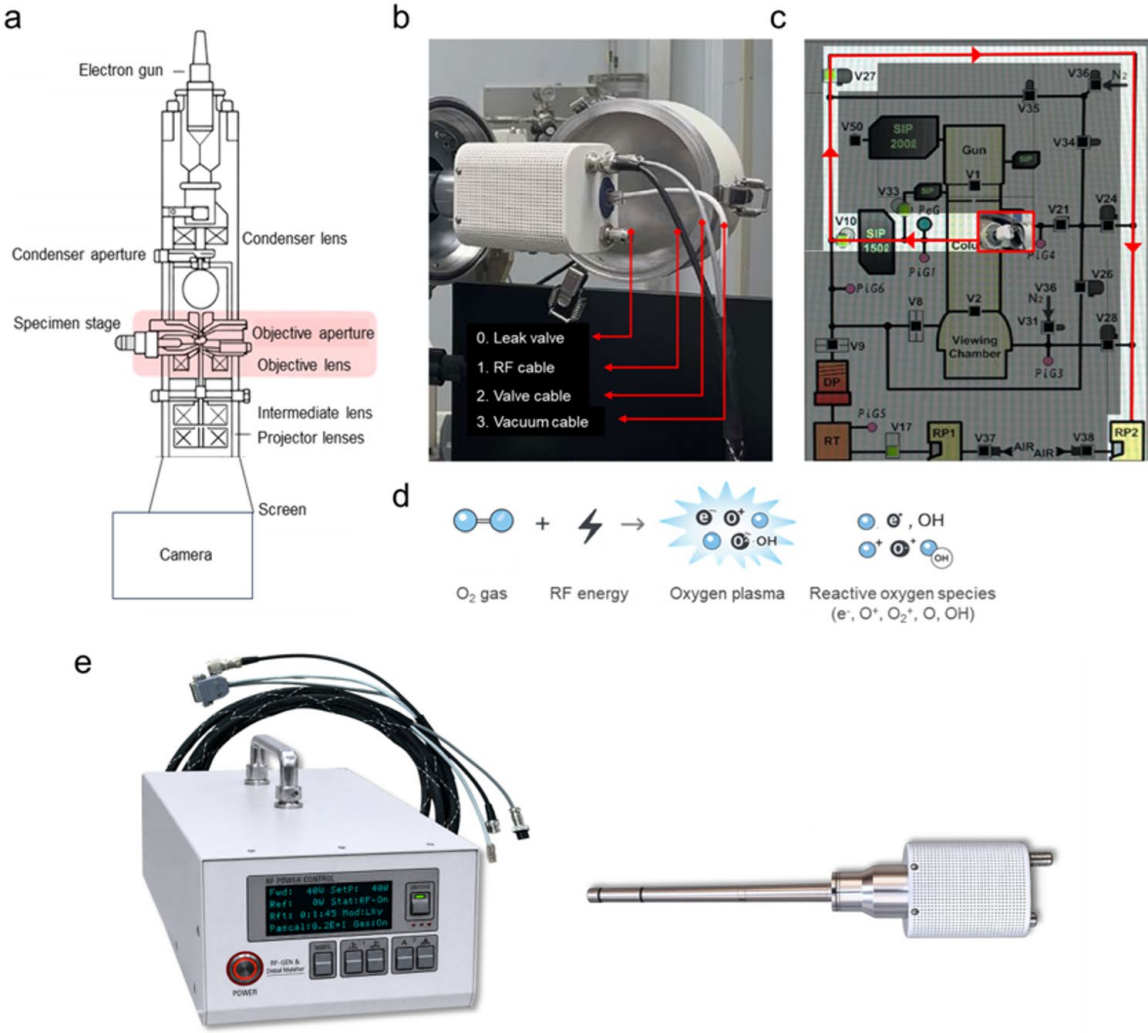
Holder-type plasma cleaning system in TEM. **a** Schematic of the TEM showing the plasma cleaning area near the specimen chamber. **b** Photograph of the plasma cleaner inserted into the TEM, with labels for the leak valve, RF cable, valve cable, and vacuum cable. **c** Diagram of the TEM vacuum system illustrating the gas flow pathway during plasma cleaning. **d** Schematic of oxygen plasma generation under RF excitation and the formation of reactive oxygen species. **e** Full-view photograph of the holder-type plasma cleaning system, showing the plasma cleaner probe, RF power supply controller, gas supply line, and connecting cables

Plasma cleaning was performed using a TEM (JEM-ARM200CF, JEOL). Figure 1 shows the holder-type plasma cleaning system integrated into the TEM column. The cleaning area is located near the specimen chamber, as depicted in Fig. 1a. A photo of the plasma cleaner inserted into the TEM is displayed in Fig. 1b, showing the leak valve, RF cable, valve cable, and vacuum gauge line connected to the rear side of the cleaner and linked to the RF power supply controller. To establish the necessary gas flow and vacuum conditions for plasma generation, the leak valve and the TEM vacuum valves were operated in a specific sequence. First, the Leak-T button on the TEM panel was set to ON, closing the internal vacuum valves (V10 and V27) and isolating the sputter ion pump from the column. Ambient air was then introduced into the column through the plasma holder’s leak valve while the chamber pressure was monitored via the column Penning gauge (PIG1). When the gauge reading reached approximately 230 µA, the Leak-T button was set to OFF to reopen V10 and V27, establishing a continuous gas flow pathway through the column to the rotary pump (RP2). The leak valve was further adjusted to maintain a steady operating pressure of approximately 80 Pa. Once the target was established, the RF generator (13.56 MHz, 37 W; 10–300 W range) was activated to initiate plasma within the column, producing reactive oxygen species that remove hydrocarbons, as illustrated in (Fig. 1d). During operation, the introduced gas and reaction by-products (primarily CO and CO2) were continuously evacuated through the vacuum system and directed to the rotary pump at the back end of the system (RP2) (Fig. 1c). Plasma cleaning was carried out for 3 h. After cleaning, the system was returned to high-vacuum conditions prior to STEM spectroscopic measurements.

### Quantitative contamination measurements using STEM-EDX and EELS

A Si lamella specimen was processed using a focused ion beam (FIB) milling system (Helios NanoLab 450 S; Thermo Fisher Scientific). STEM measurements were performed using an aberration-corrected STEM operated at 200 kV (JEM-ARM200CF, JEOL). The column vacuum during measurements was approximately 7 × 10^− 5^ Pa, measured without liquid-nitrogen cold-trap cooling. The probe current and semi-convergence angle were set to 60 pA and 24 mrad, respectively. STEM-EDX mapping was performed using a dual-type silicon drift detector (JEOL JED-2300T) (solid angle: 1.2 sr) over a field of view of 247 × 247 nm^2^ with 256 × 256 pixels and a dwell time of 0.1 ms per pixel. The resulting electron dose was approximately 4.0 × 10^4^ e^-^/nm^2^ per frame. Low-loss EELS spectra for thickness analysis were acquired under the same probe conditions with a dwell time of 2 ms per pixel and a step size of 1 nm. The spectrometer collection semi-angle was 55 mrad.

The contamination rate was quantified using two complementary metrics. The C-K signal increase rate was calculated from the STEM-EDX C-K edge intensity using Eq. (1):

1 $$\begin{aligned}C&-K signal \, increase \, rate \left(\%/frame\right) \, \\&=\, [Count\left(f\right)\, - \, Count \left(\mathit0 \right)]\, / \, [Count\left(\mathit0 \right) \, \times \, N_{frame}]\times 100\end{aligned}$$

where *Count(f)* and *Count(0)* represent the C-K counts in the final and initial frames, respectively, and *N*_*frame*_ is the total number of frames. The specimen thickness was estimated from the low-loss EELS data using the log-ratio method, *t = λ ln(I*_*t*_*/I*_*0*_*)* (Malis et al. 1988), where *I*_*t*_ is the total spectral intensity, and *I*_*0*_ is the zero-loss peak intensity.

The carbon deposition rate was then calculated using Eq. (2):

2 $$\begin{aligned}Carbon \, deposition \, rate\, \left(nm/frame\right) \, &= \, [t / \lambda \left( Contam\right) \, - \, t/ \lambda \left(Clean \right)] \, \\&\times \, \lambda_{Carbon} \, / \, N_{frame}\end{aligned}$$

where *λ*_*Carbon*_ is the inelastic mean free path of electrons in amorphous carbon (*λ*_*Carbon*_ = 123.4 nm at 200 kV and a collection semi-angle of 55 mrad). Note that prior to each measurement session, the Si lamella was plasma-cleaned using an external plasma cleaner (Fischione Model 1020, Ar/O_2_, 45 W, 5 min) to remove surface hydrocarbons and ensure that the measured contamination rates reflect chamber-originated contamination.

## Results

### Evaluation of contamination reduction

**Fig. 2:**
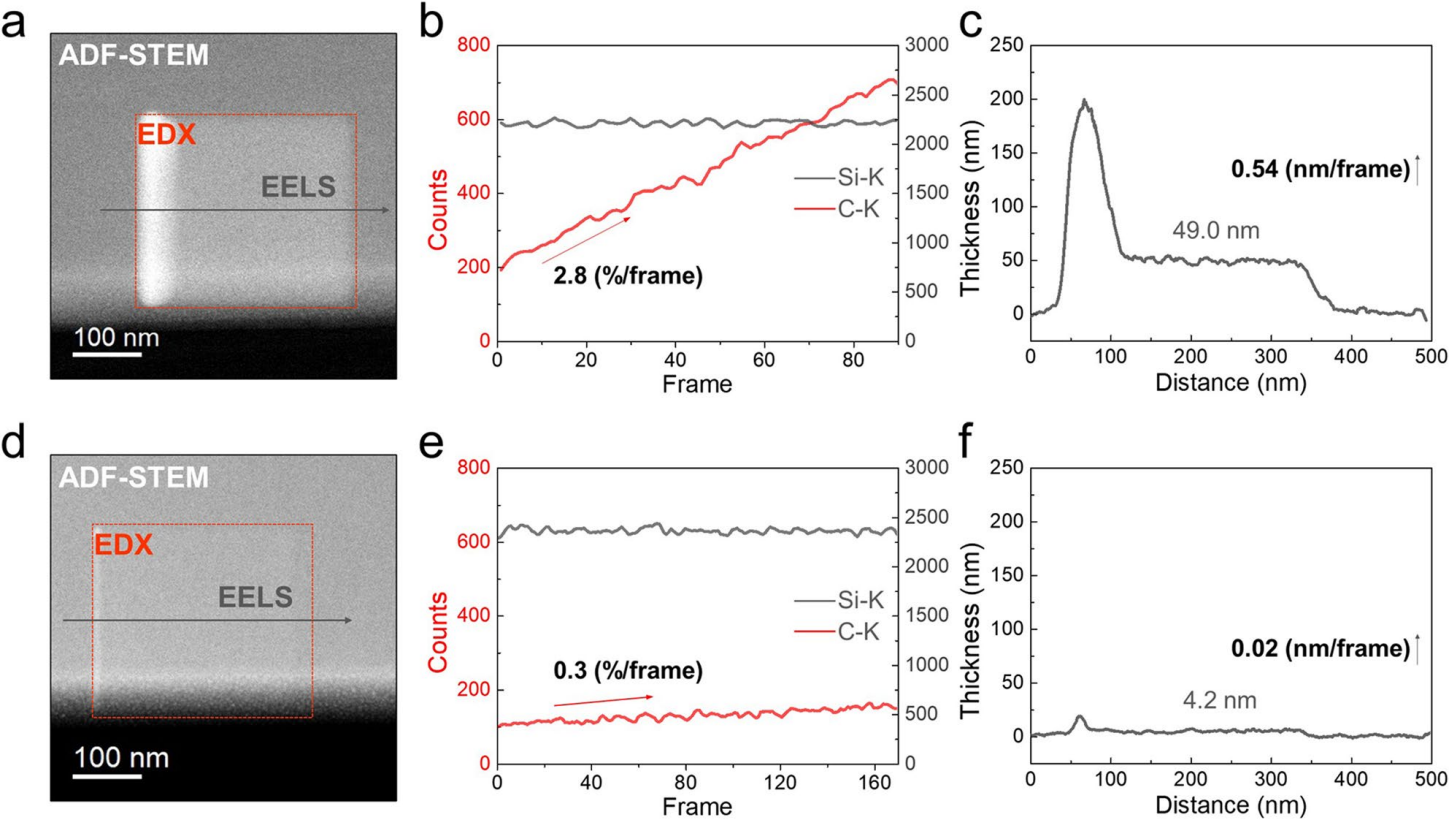
Quantitative analysis of contamination before and after chamber plasma cleaning. **a**-**c** Before cleaning and **d**-**f** after cleaning. **a**, **d** ADF-STEM images of the Si substrate. Red dashed boxes highlight the regions irradiated during STEM-EDX mapping. The contrast difference shown in the lower regions of the Si lamella in the ADF-STEM images arises from the difference in thickness and the surface-damaged layer by Ga implantation. **b**, **e** Frame-by-frame EDX counts of the C-K (0.282 keV) and Si-K (1.740 keV) edges. The C-K signal increase rates are 2.8% per frame in (**b**) and 0.3% per frame in (**e**). **c**, **f** Thickness profiles along the gray arrows in (**a**) and (**d**), respectively. Carbon deposition rates are 0.54 nm per frame in (**c**) and 0.02 nm per frame in (**f**)

To investigate the effect of plasma treatment inside the microscope, quantitative spectroscopy measurements were performed on a Si lamella specimen (Fig. 2). During prolonged STEM-EDX mapping, C-K signal intensities were recorded frame-by-frame to monitor the increase in carbon signal caused by beam-induced contamination. In addition, STEM-EELS line profiles were acquired to determine the thickness of the deposited contamination layer. The experimental conditions and the calculation procedures for contamination rates are described in Sect.  2. Before cleaning, the C-K signal increased continuously over 90 frames, at a rate of 2.8% per frame, indicating progressive accumulation of carbon contamination (Fig. 2a, b). After plasma cleaning (Fig. 2d, e), the increase in C-K signal was substantially suppressed over 170 frames, decreasing to 0.3% per frame. Meanwhile, the Si-K signal remained stable throughout the measurements, confirming that the observed change originates from carbon deposition rather than variations in the substrate signal. The EELS thickness profiles further illustrate the cleaning effect (Fig. 2c, f). Before cleaning, the thickness profile along the indicated direction exhibited a clear increase within the irradiated region, consistent with the observed C-K signal increase. The corresponding carbon deposition rate was 0.54 nm per frame. In contrast, after cleaning, the thickness increase became minimal, with a contamination thickness of only 4 nm and a deposition rate of 0.02 nm per frame. These results demonstrate that the holder-type plasma cleaner effectively reduces hydrocarbon contamination inside the chamber and suppresses beam-induced carbon deposition during STEM spectroscopy.

The thickness values shown in Fig. 2c, f represent the deposited carbon contamination thickness, obtained by subtracting the baseline Si specimen thickness measured in the adjacent non-irradiated region from the total thickness measured within the irradiated area. The relatively thick carbon line at the left edge of the scanning region corresponds to the probe fly-back position, where the electron probe dwells for a longer period between successive scan lines, resulting in enhanced local carbon deposition. It should be noted that the present experiments were performed without liquid-nitrogen cold-trap cooling in order to isolate the cleaning effect of the holder-type plasma cleaner without the additional contamination suppression provided by cryogenic trapping. Under routine operating conditions with cold-trap cooling, the baseline contamination rate is expected to be substantially lower, and the combination of plasma cleaning with cold-trap operation is anticipated to provide further enhanced contamination suppression, as the two methods address complementary aspects of hydrocarbon contamination: plasma cleaning removes hydrocarbon reservoirs from internal surfaces, while the cold trap captures mobile species from the residual gas phase.

### Time evolution of contamination rates

**Fig. 3:**
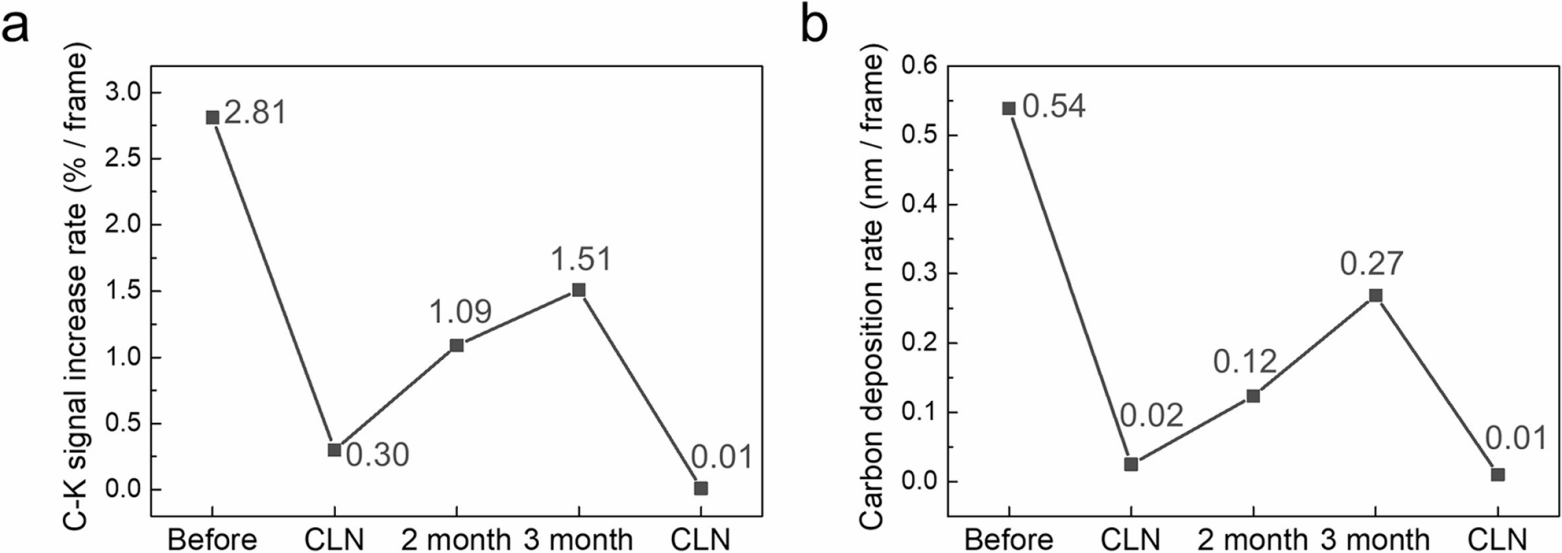
Evolution of contamination rates over time**.** Contamination levels were measured five time points across three months: before cleaning, immediately after cleaning, immediately after cleaning (CLN), two months after cleaning, three months after cleaning, and following a second cleaning cycle (CLN). **a** Rate of C-K signal increase derived from STEM-EDX data. **b** Carbon deposition rate determined from EELS thickness analysis

The persistence of the cleaning effect was further examined over a three-month period (Fig. 3). Contamination rates were measured at five time points: before cleaning, immediately after cleaning (labeled ‘CLN’), two months after cleaning, three months after cleaning, and after a second cleaning cycle. Two independent contamination metrics were evaluated: the C-K signal increase rate derived from STEM-EDX data (Fig. 3a) and the carbon deposition rate obtained from EELS thickness analysis (Fig. 3b). Both metrics exhibited consistent trends. Immediately after the first cleaning, the contamination rates decreased by approximately one order of magnitude relative to the pre-cleaning value, demonstrating the strong effectiveness of the chamber cleaning procedure. Over the following months, the contamination rates gradually increased, and after three months, the rates had returned to approximately half of the pre-cleaning level, indicating gradual accumulation of hydrocarbon species during routine microscope operation. A second cleaning cycle further reduced the contamination rates to levels below those achieved after the first cleaning (C-K signal increase rate: from 0.30 to 0.01% per frame; carbon deposition rate: from 0.02 to 0.01 nm per frame). During the three-month monitoring period, the TEM was operated daily for routine STEM imaging and spectroscopy of various specimens, ranging from low-dimensional nanomaterials and transition metal oxides to metal alloys and micro fuel cell electrodes. Liquid-nitrogen cold-trap cooling was used during routine operation. No major maintenance procedures, such as column venting or aperture replacement, were carried out during this period.

## Discussion

The observed reduction in contamination rates after plasma cleaning can be attributed to the generation of reactive oxygen species within the column, which oxidize hydrocarbon species adsorbed on internal surfaces, converting them into volatile products such as CO and CO_2_ that are continuously evacuated by the vacuum system. The mechanism is consistent with previous reports on plasma-assisted hydrocarbon removal (Isabell et al. 1999; Tang et al. 2023). However, the present configuration enables these reactions to occur directly inside the TEM chamber in approximately 3 h without disassembly, offering a substantial time advantage over conventional column baking procedures, which typically require several days of microscope downtime. This difference in turnaround time has practical implications for facilities with high instrument utilization, where extended maintenance windows are difficult to schedule.

The cleaning effectiveness observed in this study, an approximately one-order-of-magnitude reduction in both contamination metrics, is comparable to levels achieved by holder bakeout systems (Goh et al. 2020) and to the contamination suppression reported after combined plasma cleaning and cold-trap operation (Mitchell 2015). What is noticeable in the present work is the quantitative tracking of contamination recovery over a three-month period, which provides practical guidance for determining cleaning intervals. The gradual return of contamination rates to approximately half of pre-cleaning levels over three months suggests continuous reintroduction of hydrocarbons during routine operation, likely originating from specimen transfer, holder components, and residual gas adsorption (McGilvery et al. 2012; Hugenschmidt et al. 2023). The observation that a second cleaning cycle reduced contamination rates below those achieved after the first cleaning (C-K signal increase rate: from 0.30 to 0.01%/frame) suggests a cumulative benefit of repeated cleaning. One possible explanation is that the first cycle removes loosely adsorbed hydrocarbons from accessible surfaces, while the second cycle addresses more strongly bound residues or species that re-adsorbed from regions not fully reached during the initial treatment. This cumulative effect indicates that repeated cleaning cycles may progressively condition the internal chamber environment, leading to sustained improvements in contamination performance.

Some limitations of the present study should be noted. The experiments were performed on a single instrument (JEOL JEM-ARM200CF), and the effectiveness of the holder-type plasma cleaner on other TEM platforms remains to be verified. Additionally, the present work used ambient air as the process gas for convenience, but the optimal gas composition for maximizing cleaning efficacy while minimizing instrument damage has not been systematically investigated. Regarding the potential impact of oxygen plasma on internal TEM components, the RF power level used in this study (37 W) produces low-energy reactive oxygen species that primarily react chemically with hydrocarbon species rather than causing physical sputtering. The ion energies in this regime remain well below the sputtering threshold for the metallic and ceramic components of the TEM column (Isabell et al. 1999). Over the course of multiple cleaning cycles spanning several months, no measurable degradation in imaging resolution or spectroscopic performance was observed.

## Conclusions

In this report, we demonstrated a holder-type plasma cleaning method for removing hydrocarbon contamination from within a TEM chamber. By generating reactive oxygen species under RF excitation, contamination rates were reduced by about one order of magnitude within three hours, as confirmed by quantitative STEM-EDX and EELS analyses. The cleaning effect gradually diminished over three months of routine operation but remained well below pre-cleaning levels. Additionally, repeated cleaning cycles further enhanced contamination suppression, showing the effectiveness of this approach as a periodic maintenance strategy. These results establish holder-type plasma cleaning as an effective and practical method for improving the reliability of high-resolution imaging and quantitative spectroscopy in STEM.

## Data Availability

The datasets used and/or analyzed during the current study are available from the corresponding author on reasonable request.
